# Prokaryotic expression and mechanism of action of α-helical antimicrobial peptide A20L using fusion tags

**DOI:** 10.1186/s12896-015-0189-x

**Published:** 2015-08-05

**Authors:** Tonghui Yi, Shiyu Sun, Yibing Huang, Yuxin Chen

**Affiliations:** Key Laboratory for Molecular Enzymology and Engineering of the Ministry of Education, Jilin University, 2699 Qianjin St., Changchun, Jilin 130012 P. R. China; National Engineering Laboratory for AIDS Vaccine, Jilin University, Changchun, China; School of Life Sciences, Jilin University, Changchun, China

**Keywords:** Antimicrobial peptide, Ubiquitin, Small ubiquitin-related modifier, Liposome, Specificity

## Abstract

**Background:**

Antimicrobial peptides have become important candidates as new antibiotics against resistant bacterial strains. However, the major industrial manufacture of antimicrobial peptides is chemical synthesis with high costs and in relatively small scale. The Ub-tag and SUMO-tag are useful for increasing the yield of enzymes and other proteins in expression system. In this study, antimicrobial peptide A20L (KWKSFLKTFKSAKKTVLHTLLKAISS), a derivative of V13K in the previous study is used as a template to be expressed in different Ub-tag and human SUMO tag systems to compare the prokaryotic expression approaches of antimicrobial peptide. The antibacterial mechanism of action and membrane specificity of A20L was further studied.

**Methods:**

We fused the Ub and SUMO1/2/3/4 with A20L to construct expression plasmids. Ub-A20L and SUMO1/2/34 gene sequences were inserted into the pHUE plasmids and pET-28b+ plasmids, respectively, to construct pHUE-A20L plasmids and pET-28b+-SUMO1/2/3/4-A20L plasmids. These plasmids were transformed into *E. coli* Rosetta (DE3) and induced with IPTG to express Ub-A20L and SUMO1/2/3/4 fusion proteins. The recombinant proteins were found in the soluble fraction after being over expressed in *E. coli* Rosetta (DE3). Antibacterial and hemolytic activities and membrane permeabilization ability of A20L were determined. Peptide structure was also studied by circular dichroism experiments.

**Results:**

A20L (KWKSFLKTFKSAKKTVLHTLLKAISS) was successfully expressed by fusion with an ubiquitin tag (Ub-tag) and human SUMO tags (SUMO1/2/3/4-tags). A20L exhibited antimicrobial activity against various Gram-negative and Gram-positive bacteria. Based on the hemolytic activity against human red blood cells, A20L showed good specificity against bacteria. The circular dichroism experiments illustrated that A20L was transferred into an α-helical structure in the presence of hydrophobic environment. The antibacterial mechanism of action and membrane specificity of A20L was further studied using membrane permeabilization experiments and tryptophan fluorescence and quenching experiments in liposomes.

**Conclusions:**

The Ub-tag and human SUMO-tags represent good alternatives to chemical synthesis for the industrial production of antimicrobial peptides with low costs and high yields. The antibacterial mechanism of action of A20L was proved as membrane disruption. A20L showed stronger specificity on liposomes mimicking bacterial membrane than those mimicking eukaryotic cell membrane, which is consistent with the biological activity studies.

**Electronic supplementary material:**

The online version of this article (doi:10.1186/s12896-015-0189-x) contains supplementary material, which is available to authorized users.

## Background

Bacteria are developing resistance to antibiotics at an alarming rate, thus the development of new antibiotic alternatives continues to be extremely urgent [1, 2]. Because of their antibacterial properties, antimicrobial peptides (AMPs) are expected to become ideal antibiotic alternatives [3]. AMPs are generally between 10 and 50 amino acid residues, and are strongly cationic (pI 8.9–10.7) [4]. AMPs are characterized by their broad spectrum of activities and their antibacterial mechanism differs from traditional antibiotics [5]. To date, there are more than 2000 types of AMPs that have been isolated, including some that have been used in the health care and food processing industries [6, 7].

AMPs are primarily harvested via natural extraction, chemical synthesis and genetic engineering. The large-scale application of AMPs is restricted by limitations in sourcing the natural products and the high production costs of chemical synthesis. A prokaryotic expression system can reduce the production cost of AMPs and increase their large-scale applications [8]. However, prokaryotic expression of AMPs is technically challenging because AMPs are not only toxic to the host, but also susceptible to exogenous protease degradation. Previous studies have shown that use of a fusion tag system can achieve effective expression of AMPs [9]. Fusion tags commonly used include thioredoxin, steroid isomerase and glutathione-S-transferase enzymes. However new fusion tags useful to improve the yield of AMPs are continuously coming out.

Ubiquitin (Ub) has 76 amino acids and mainly functions to label proteins for hydrolysis [10]. Small ubiquitin-related modifier (SUMO) consists of about 100 amino acid residues, and modulates protein structure and function by covalent modification of target proteins in eukaryotes. Yeast cells have only one SUMO gene (SMT3), while there are four SUMO genes in vertebrates, SUMO1, SUMO2, SUMO3, SUMO4 [11]. Recent studies have shown that Ub and SUMO were used as fusion tags. Their small sizes bring relatively high peptide-to-carrier ratios, favors the increase of yield in the production of antimicrobial peptides. The highly specific sumoase facilitates efficient release of the antimicrobial peptide [12]. In previous studies, we obtained the AMP A20L (KWKSFLKTFKSAKKTVLHTLLKAISS, 2991.6 Da) [13], which is a derivative of the V13K in which the amino acid alanine in position 20 was substituted by leucine, and showed that it possesses good antimicrobial activity and low hemolytic activity. In this study, we used the Ub-tag and human SUMO1/2/3/4-tag fusion to express A20L in a prokaryotic system and further explore the antibacterial mechanism of action of AMPs with membrane permeabilization experiments, tryptophan fluorescence and quenching experiments in liposomes.

## Methods

### Materials

*Escherichia coli* (*E. coli*) strain DH5α, Rosetta (DE3) and the expression plasmid pET-28b + were purchased from Novagen Co. Ltd. (Darmstadt, Germany). Sumoase and pHUE were gifts from Prof. Xuexun Fang, Jilin University, China. The plasmid pHUE was constructed for the expression of His-tagged ubiquitin (Ub) fusion proteins by modifying pET15b. It contains the inducible T7 RNA polymerase promoter, a histidine tag at the 5′ end of an ubiquitin open reading frame and an extended polylinker [14]. pUC57-SUMO1/2/3/4-A20L and pUC57-A20L plasmids were constructed by Genewiz Co. Ltd. (Suzhou, China). *E. coli* ATCC25922, *Pseudomonas aeruginosa* (*P. aeruginosa*) ATCC27853, *Staphylococcus aureus* (*S. aureus*) ATCC25923, *Bacillus subtilis* (*B. subtilis*) ATCC6633 and *E. coli ML-35* ATCC 43827 were purchased from the American Type Culture Collection (USA). Restriction endonucleases *Nde*I, *Bam*HI and *Eco*RI were purchased from Takara Biotech Co. Ltd. (Dalian, China). The bicinchoninic acid (BCA) protein assay kit was purchased from Shanghai Biological Engineering Co., Ltd., China. Luria-Bertani (LB) medium and Mueller-Hinton (MH) medium were purchased from Dingguo Co. Ltd. (Beijing, China). CM Sepharose FF chromatography medium was purchased from GE Chemical Co. (Uppsala,USA), and Ni-NTA chromatography medium was purchased from Qiagen Co. Ltd. (Hilden, Germany). Prestained SDS-PAGE standards were purchased from Transgen Co. Ltd. (Beijing, China). Trifluoroethanol (TFE), 1-N-phenylnaphthylamine (NPN), o-nitrophenyl-β-D-galactoside (ONPG) and phenylmethanesulfonyl fluoride (PMSF) were purchased from Sigma (Beijing, China). L-α-phosphatidyl-dl-glycerol (PG), L-α-phosphatidylcholine (PC) and cholesterol were purchased from Avanti Polar Lipids, Inc. (Alabama, USA). HEPES and KI were purchased from Beijing Chemical Works (Beijing, China).

### Plasmid construction

**Fig. 1:**
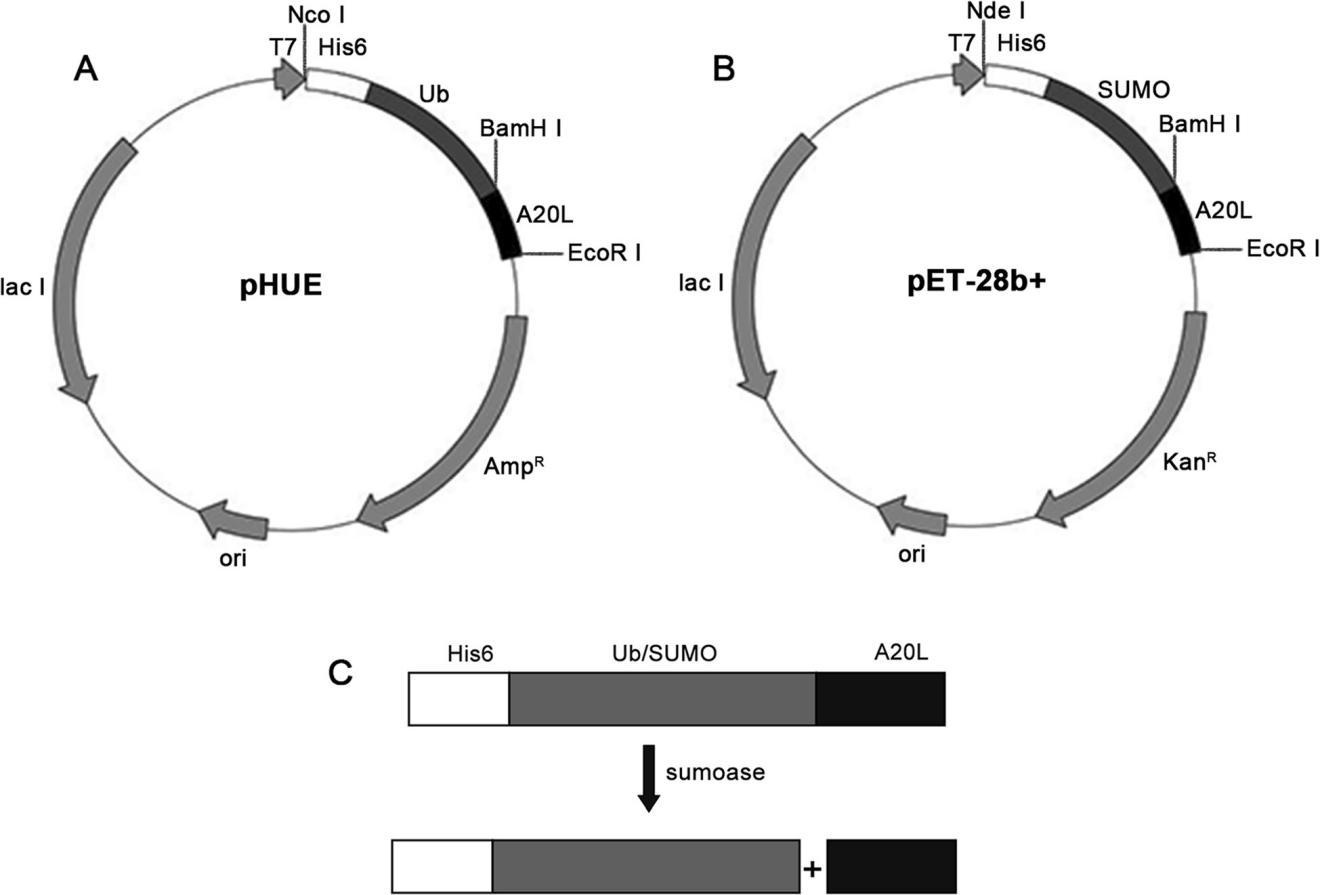
The cloning and cleavage strategy for Ub-A20L and SUMO1/2/3/4-A20L. (**a**) pHUE-A20L, which was constructed by inserting the A20L gene at the *Bam*HI/*Eco*RI sites of pHUE; (**b**) pET28b + −SUMO1/2/3/4-A20L, which was constructed by inserting the A20L gene at the *Bam*HI/*Eco*RI sites of pET-28b+; (**c**) the cleavage strategy for Ub-A20L and SUMO-A20L

The pUC57-SUMO1/2/3/4-A20L and pUC57-A20L plasmids were served as templates. The A20L gene sequence (AAATGGAAATCTTTCCTGAAAACCTTCAAATCTGCTAAAAAAACCGTTCTGCACACCCTGCTGAAAGCTATCTCTTCT) was amplified by PCR (Additional file 1: Table S1 and Table S2). The PCR product of the A20L and pHUE plasmid was doubly digested by *Bam*HI and *Eco*RI. The PCR products of SUMO1/2/3/4-A20L and the pET-28b + plasmid were doubly digested with *Nde*I and *Eco*RI. After digestion, T4 DNA Ligase was used to link and transform *E. coli* DH5α competent cells, and then monoclonal colonies were inoculated in LB culture medium for plasmid sequencing. The plasmid construction process is shown in Fig. 1.

### Fusion protein expression, purification and digestion

The pET-28b + −SUMO1/2/3/4-A20L and pHUE-A20L plasmids were transformed into *E. coli* Rosetta (DE3) competent cells. Single colonies were picked, shaken and cultured at 37 °C and 180 rpm for 16 h. Then, the bacterial suspension at a volume ratio of 1:100 was cultured in LB medium, shaken at 37 °C, 180 rpm for 3 h until the OD_600 nm_ was 0.4–0.6. Isopropyl β-D-1-thiogalactopyranoside (IPTG) at a final concentration of 1 mM was added to induce expression at 32 °C and 180 rpm for 4 h followed by centrifugation (6000 × g for 10 min). After addition of Buffer A (50 mM Na_2_HPO_4_/NaH_2_PO_4_, 300 mM NaCl, 1 mM phenylmethanesulfonyl fluoride, pH 7.4), the samples were sonicated (200 W, 10 min) and centrifuged to collect the supernatant. A 2 mL Ni-NTA column was washed and equilibrated with Buffer B (50 mM Na_2_HPO_4_/NaH_2_PO_4_, 300 mM NaCl, pH 7.4) and then the supernatant was cycled over the Ni-NTA column for 1 h. After rinsing with Buffer B and 10 mL Buffer C (50 mM Na_2_HPO_4_/NaH_2_PO_4_, 300 mM NaCl, 50 mM imidazole, pH 7.4), 10 mL Buffer D (50 mM Na_2_HPO_4_/NaH_2_PO_4_, 300 mM NaCl, 300 mM imidazole, pH 7.4) was used to elute the target proteins, Ub-A20L and SUMO1/2/3/4-A20L. Target proteins were dialyzed against 500 mL Buffer E (20 mM Na_2_HPO_4_/NaH_2_PO_4,_ pH 8.4) overnight. A 10 mL CM Sepharose FF column was first equilibrated with Buffer E, and loaded with the target proteins for 1 h, followed by purification with Buffer F (20 mM Na_2_HPO_4_/NaH_2_PO_4_, 0.1–1 M NaCl, pH 8.4) to yield Ub-A20L and SUMO1/2/3/4-A20L. The purity of the target proteins was assessed using 12 % SDS-PAGE electrophoresis. The fused proteins were combined with sumoase at a molar ratio of 1:50 and digested overnight at 4 °C. After digestion, a Sephadex G25 column (2 cm × 80 cm) was employed for separation purpose. Buffer G (20 mM Na_2_HPO_4_/NaH_2_PO_4,_ pH 7.4) was used as an eluent to purify A20L. Tricine SDS-PAGE electrophoresis analysis was used to check the purity of A20L, and the polypeptide yield was determined using the Walker method [15]. The concentrations of A20L were calculated in the equation: A20L (in μg/ml) = 144 × (OD_215nm_- OD_225nm_). The experiments were separately performed six times.

### Antibacterial activity determination

The minimal inhibitory concentration (MIC) of A20L was determined using the broth dilution method [16]. A single bacterial colony was picked and cultured at 37 °C, 180 rpm for overnight. Wells of a 96-well plates were filled with 90 μL of the bacterial suspension (5 × 10^5^ CFU/mL in MH media) and 10 μL of of serial two fold dilutions of the A20L peptide (stock concentration 2 mg/mL) and cultured at 37 °C and 170 rpm, for 24 h. The OD_590 nm_ was determined to calculate the MIC. The experiments were performed in triplicates.

### Hemolytic activity determination

The A20L peptides were doubly diluted at an initial concentration of 2 mg/mL and added to 96-well plates at a density of 70 μL per well. Healthy human blood (1–2 mL) from healthy volunteers was centrifuged at 1000 × g for 5 min and washed thrice with PBS. Erythrocytes were resuspended and counted, and diluted to a concentration of 2 × 10^8^ cells/mL. The diluted erythrocytes were added to 96-well plates at a density of 70 μL per well and mixed with A20L, incubated at 37 °C for 2 h, and centrifuged at 3000 × g for 10 min. The release of hemoglobin was determined by measuring the optical density of the supernatant at OD_578 nm_. The hemolytic activity was measured as the minimal hemolytic concentration (MHC). PBS containing 1 % erythrocytes and distilled water were used as a negative control and 100 % hemolysis positive control [16], respectively. The experiments were performed in triplicates.

### Circular dichroism experiments

The secondary structures of the A20L peptide at a concentration of 75 μM were determined using a Jasco J-810 circular dichroism spectrometer (Jasco, Easton, MD) at 25 °C in non-denaturating buffer (100 mM KCl, 50 mM KH_2_PO_4_/K_2_HPO_4,_ pH 7.0) or in the presence of α-helix inducing 50 % 2,2,2-trifluoroethanol (TFE) (non-denaturating buffer:TFE = 1:1, *vol/vo*l). The A20L secondary structure was confirmed by calculating the average molar ellipticity at 222 nm ([*θ*]_222_) [17]. The experiments were separately performed three times.

### Outer membrane permeabilization experiments

The change of outer membrane permeability in bacteria after interaction with A20L was analyzed using the hydrophobic fluorescent probe 1-N-phenylnaphthylamine (NPN). *P. aeruginosa* was inoculated in 20 mL LB liquid medium, shaken and cultured at 37 °C for 18 h. A 1 mL bacterial suspension was inoculated into 50 mL of LB medium, shaken and cultured at 37 °C until the OD_600 nm_ was 0.4–0.6. After centrifugation (4000 × g for 10 min), bacteria were collected and resuspended in buffer (5 mM HEPES, 5 mM NaN_3_, 0.25 mM NPN, pH 7.4) to OD_600 nm_ = 0.5 which equated to 300 μL of a 800 μg/mL A20L solution added to 2.7 mL of bacterial suspension. A negative control was prepared in the same volume of reaction buffer (5 mM HEPES, 5 mM NaN_3,_ pH 7.4). A Shimadzu RF-5301 PC spectrofluorophotometer (excitation wavelength 350 nm, emission wavelength 420 nm) was used to continuously collect data for 10 min [18]. The experiments were separately performed ten times.

### Inner membrane permeabilization experiments

Permeabilization of the inner membrane was assessed by measuring the access of ο-nitrophenyl β-D-galactopyranoside (ONPG) to the cytoplasm. We used the permease-deficient strain *E. coli ML-35*, which constitutively expresses cytoplasmic β-galactosidase. *E. coli ML-35* was cultured in LB medium containing 5 % lactose. The bacterial cells were collected and resuspended with sterile water to an OD_420nm_ of 1.2. A total of 1000 μL of bacterial suspension was mixed with 100 μL of 30 mM o-nitrophenyl-β-D-galactosidase (ONPG), and then added to 900 μL of 16 μg/mL A20L. The negative control was carried out with 0.5 % NaCl. A Shimadzu UV-2550 UV spectrophotometer (wavelength set to 420 nm) was used to continuously collect data for 90 min [19].

### Tryptophan fluorescence and quenching experiments

As reported previously, the liposomes were prepared and cultured in HEPES buffer (pH 7.4, 10 mM HEPES, 150 mM NaCl) such that their molar concentration was adjusted to 100 μM [20]. A total of 14 μL of 100 μM A20L and 686 μL of 100 μM liposomes were combined together at 25 °C for 10 min. A Shimadzu RF-5301 PC, (excitation wavelength: 350 nm, emission wavelength of 300–450 nm) was used to measure the tryptophan fluorescence [20]. Potassium iodide (KI) at a final concentration of 0.02–0.08 M was added to the reaction system and the tryptophan fluorescence was measured as described above. The Stern-Volmer equation was used to calculate the quenching constant K_sv_*.* The experiments were performed in triplicates.

### Ethics and consent statements

Research involving human subjects that is reported in the manuscript has been performed with the approval of the ethics committee of School of Life Sciences, Jilin University, China (Reference No. JSK-RTLL2014003). Research carried out on humans is in compliance with the Helsinki Declaration. The written informed consent for the participant who supplies human red blood cells in this study was obtained from the participant.

## Results

### Expression, purification and digestion of Ub-A20L and SUMO1/2/3/4-A20L

**Fig. 2:**
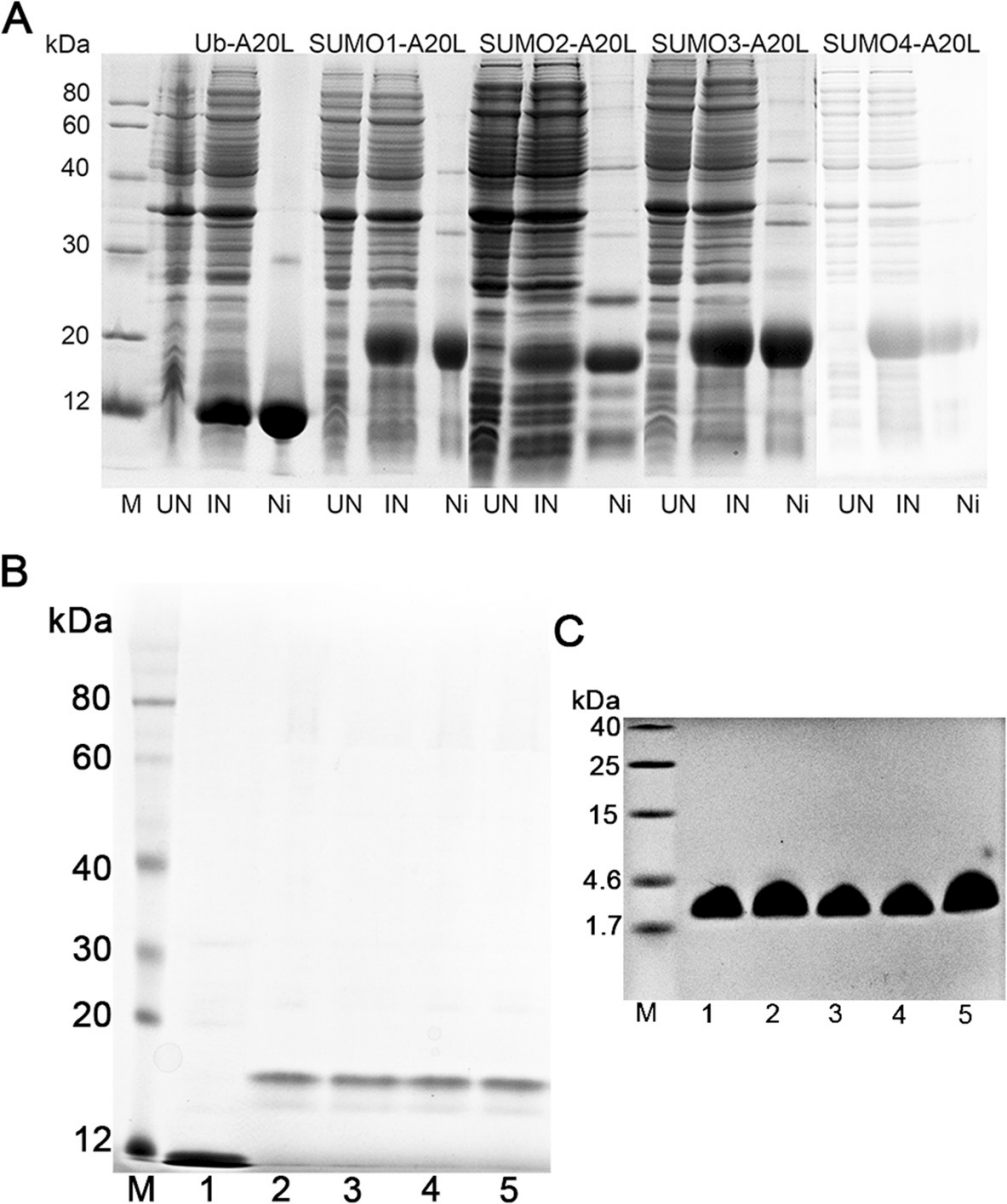
(**a**) Expression of A20L fused with Ub, SUMO1, SUMO2, SUMO3 and SUMO4 proteins. (M): molecular weight marker; (UN): un-induced culture; (IN): induced culture; (Ni): Ni-NTA purified pool; (**b**) purification of Ub-A20Land SUMO1/2/3/4-A20L by CM Sepharose FF. (M): molecular weight marker; (1) Ub-A20L; (2) SUMO1-A20L; (3) SUMO2-A20L; (4) SUMO3-A20L; (5) SUMO4-A20L; (**c**) purification of A20L (M): molecular weight marker; (1–5): A20L purified from Ub-A20L(1),SUMO1-A20L(2),SUMO2-A20L(3), SUMO3-A20L(4),SUMO4-A20L(5)

To explore the function of Ub protein and SUMO1/2/3/4 proteins as fusion tags, we fused the Ub and SUMO1/2/3/4 with A20L to construct expression plasmids. Ub-A20L and SUMO1/2/34 gene sequences were inserted into the pHUE plasmids and pET-28b + plasmids, respectively, to construct pHUE-A20L plasmids and pET-28b + −SUMO1/2/3/4-A20L plasmids. These plasmids were transformed into *E. coli* Rosetta (DE3) and induced with IPTG to express Ub-A20L and SUMO1/2/3/4 fusion proteins. The recombinant proteins were found in the soluble fraction after being over expressed in *E. coli* Rosetta (DE3). Their relative expression levels were calculated by gel scanning (Fig. 2a and b). The yield of the protein expression was 76.3 mg/L and 51.5–21.9 mg/L for Ub-A20L and SUMO1/2/3/4, respectively (Fig. 2a; Table 1). After cell disruption, the supernatant was obtained and purified using Ni-NTA affinity chromatography and CM Sepharose FF cation exchange chromatography. The peptide purity was determined by 12 % SDS-PAGE, and the protein yield was determined by the BCA method. The yield of Ub-A20L and SUMO1/2/3/4-A20L was 51.2 mg/L and 13.6–36.4 mg/L, respectively (Fig. 2a, b; Table 1). After sumoase cleavage, the A20L peptide was obtained from Sephadex G25 column purification. The yield of the A20L peptide was 7.3 mg/L from Ub-A20L and 2.0–5.4 mg/L from SUMO1/2/3/4-A20L after enzyme digestion (Fig. 2c and Table 1). This indicates that both Ub and SUMO1/2/3/4 tags are able to promote the expression of the A20L peptide, but Ub-tag was effective than SUMO1/2/3/4.

**Table 1:** Purification of A20L from *E. coli* Rosetta (DE3) cells expressing Ub-A20L and SUMO1/2/3/4-A20L in a batch culture of 1 L

Protein	Target protein after 1 mM IPTG induction (mg)^a^	Ni-NTA pool (mg)^b^	CM Sepharose FF pool (mg)^b^	A20L (mg)^c^
His_6_-Ub-A20L	76.3	51.2	46.5	7.3
His_6_-SUMO1-A20L	52.0	33.8	30.6	5.4
His_6_-SUMO2-A20L	48.7	29.7	24.1	3.2
His_6_-SUMO3-A20L	61.5	36.4	32.9	4.8
His_6_-SUMO4-A20L	21.9	13.6	10.2	2.0

^a^The quantity of target protein was estimated by SDS gel scanning

^b^The quantity of target protein was determined by the BCA method

^c^The quantity of A20L was determined by the Walker method [15]

### Antibacterial and hemolytic activities

The geometric mean (GM) of the MIC values for the four microbial strains was calculated as an overall indicator of the antibacterial activity of the AMP. A20L exhibited strong antimicrobial activity against various Gram-negative and Gram-positive bacteria. The MIC values for Gram-negative and Gram-positive bacteria were 2.67–21.33 μg/mL, and the GM value was calculated to be 6.87 μg/mL, (Table 2). The MHC value of the peptide against human erythrocytes was determined to represent peptide toxicity. A20L showed negligible hemolytic activity against human red blood cells (213.33 μg/mL). The therapeutic index (TI) is a widely used parameter indicating the specificity of AMPs, and is expressed as the ratio of MHC/MIC. In this study, the TI was 31.05, indicating that the A20L peptide has high antibacterial specificity (Table 2).

**Table 2:** Antimicrobial (MIC) and hemolytic (MHC) activities of A20L

Peptide	MIC					MHC (μg/ml)	TI^b^
	(μg/ml)						
	*Escherichia Coli*	*Pseudomonas aeruginosa*	*Staphylococcus aureus*	*Bacillus subtilis*	GM^a^		
	ATCC25922	ATCC27853	ATCC25923	ATCC6633			
A20L	2.67	7.33	21.33	5.33	6.87	213.33	31.05

^a^GM denotes the geometric mean of the MIC values from all four microbial strains tested

^b^Therapeutic index (TI) = MHC (μg/ml)/geometric mean of MIC (μg/ml)

### Peptide secondary structure

**Fig. 3:**
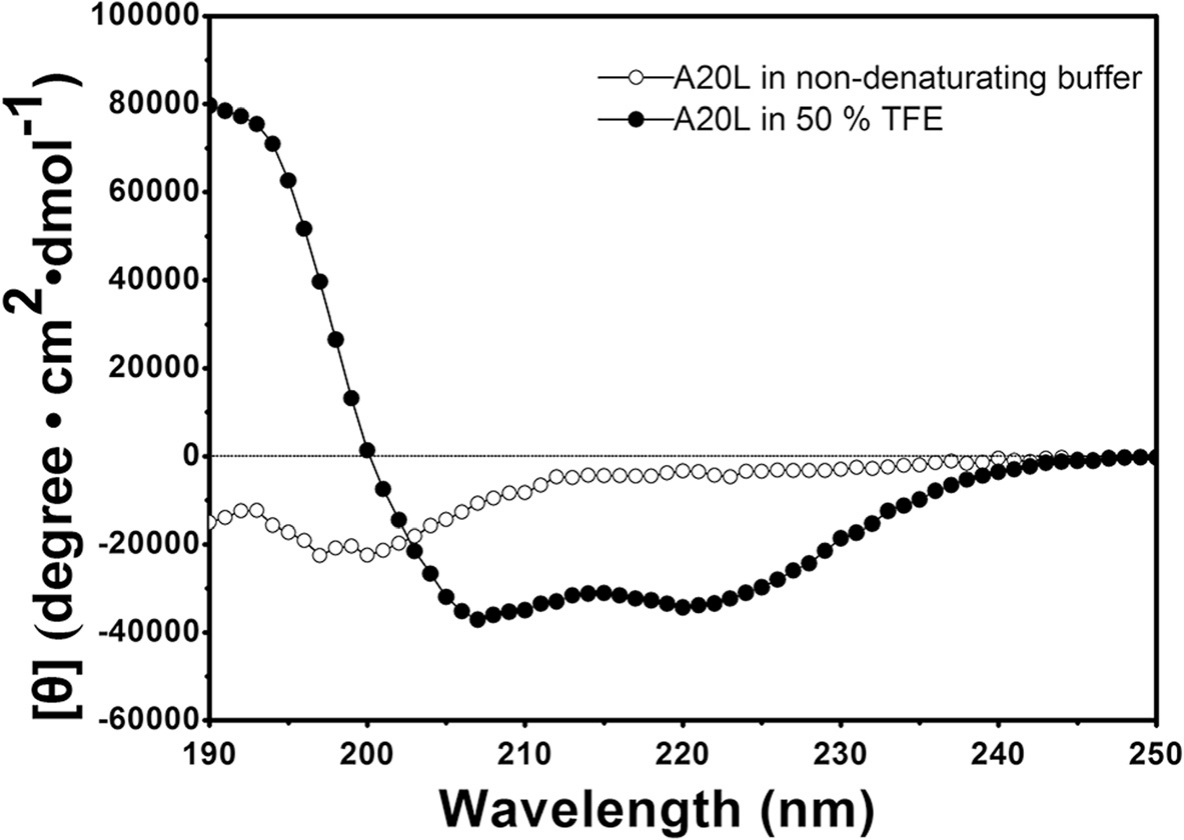
Circulat dichroism (CD) spectra of the antimicrobial peptide A20L. Open symbols denote the CD spectra of the peptide in non-denaturating conditions (KP buffer: pH 7, 50 mM KH_2_PO_4_/K_2_HPO_4_, 100 mM KCl); solid symbols denote the CD spectra obtained in the presence of an α-helix inducing solvent TFE (KP buffer: TFE = 1:1, *vol/vol*)

The helicity is a biophysical parameter closely related to the antibacterial activity [20]. In this study, we studied the variation in the secondary structure of the A20L peptide by measuring its circular dichroism spectrum under different buffer systems. The results showed that the [*θ*] _222_ of A20L in the non-denaturating buffer was −4363 degree • cm^2^• dmol^−1^, indicating that the structure of A20L is disordered, while the [*θ*] _222_ of the peptide was −33424 degree•cm^2^•dmol^−1^ in the presence of 50 % TFE to mimic the hydrophobic environment, indicating that the peptide formed a typical α-helical structure under hydrophobic environments such as biomembranes (Fig. 3).

### Membrane permeabilization

**Fig. 4:**
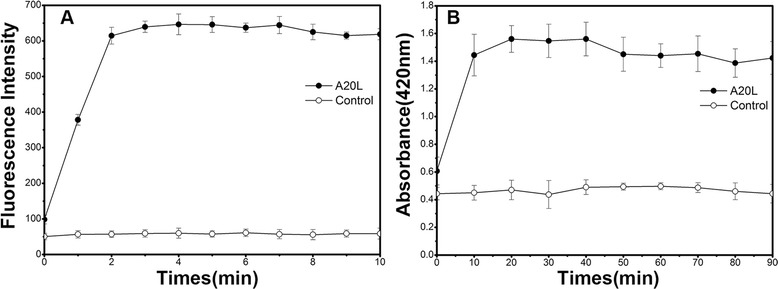
Membrane permeabilization assay of the antimicrobial peptide A20L. (**a**) outer membrane permeabilization induced by A20L was detected by NPN uptake in *P. aeruginosa* ATCC 27853; (**b**) the effect of A20L on the inner membrane of *E. coli ML-35*. Release of cytoplasmic β-galactosidase activity (measured from the OD_420 nm_) from *E. coli ML-35* treated with A20L

Gram-negative bacterial cell membranes are divided into outer and inner membranes, whilst, Gram-positive bacteria only have cytoplasmic membranes, which are composed of similar lipid components as the inner membranes of Gram-negative bacteria. *Pseudomonas aeruginosa* and *Escherichia coli ML-35* were selected for the outer membrane and inner membrane assays, respectively, since *Pseudomonas aeruginosa* is a most common infectious bacterium in clinics and *Escherichia coli ML-35* is a lactose permease-deficient strain. AMPs provoke the disruption of the bacterial membrane to kill the bacteria. After A20L interacts with bacteria, changes in the outer membrane permeability can be detected using the fluorescence probe NPN. The fluorescence intensity emitted from NPN is weak in a hydrophilic environment, but very strong in a hydrophobic environment such as the hydrophobic core of a bacterial cell membrane [18]. In the outer membrane permeabilization experiment, after the interaction of A20L with bacteria, NPN was deeply inserted into the core of hydrophobic region within 2 min, and the fluorescence intensity was observed to increase from 50 to 650 (Fig. 4a).

β-Galactosidase is located in the bacterial cytoplasm, and can hydrolyze ONPG into galactose and *o*-nitrophenol which shows yellow color. In untreated *E. coli*, ONPG is transferred into the cytoplasm via lactose permease, while if inner membrane destruction has occurred, ONPG can rapidly enter into the bacteria to react with β-galactosidase. By measuring the change in OD_420 nm_, we could determine whether or not ONPG was able to penetrate the cytoplasm, thus indicating whether the bacterial inner membrane is damaged [19]. The intracellular release of β-galactosidase became stable after 10 min and the OD_420 nm_ increased from 0.6 to 1.4 (Fig. 4b). Outer and inner membrane permeabilization assays indicate that the A20L peptide exhibited destructive effects on both bacteria membranes. Additionally, there was a clear distinction between the penetration time of A20L into the outer and inner bacterial membranes. As expected, A20L first damaged the outer membranes, then the inner membranes were also damaged subsequently.

### Interaction of A20L with liposomes

**Fig. 5:**
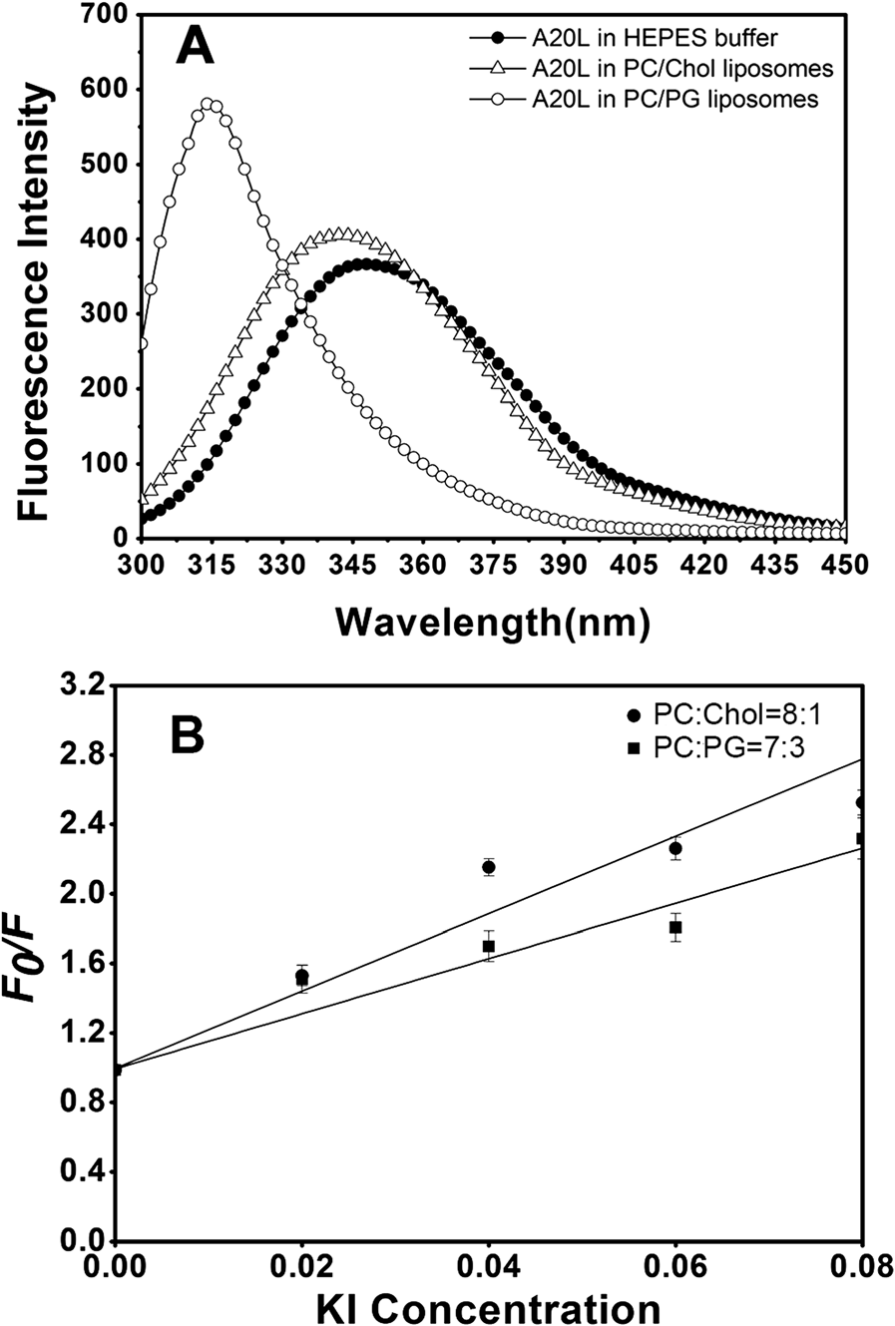
Fluorescence emission spectra and Stern-Volmer plot of A20L using various liposome models. (**a**) tryptophan fluorescence emission spectra of the antimicrobial peptide A20L; (**b**) Stern-Volmer plots of the tryptophan fluorescence were obtained by the sequential addition of the fluorescence quencher KI

In this study, tryptophan fluorescence and quenching experiments were carried out to study the insertion of A20L into different types of membrane structures. Tryptophan fluorescence Intensity is increased in a hydrophobic environment, and the blue shift occurred at the maximum emission wavelength. In the absence of the peptide, the maximum emission wavelength of tryptophan fluorescence was 346.7 nm. The liposomes PC/PG (7:3, w/w) and the PC/cholesterol (8:1, w/w) were made to mimic a bacterial and a eukaryotic membrane, respectively [21]. When the A20L interacted with the PC/cholesterol liposomes, the maximum emission wavelength was 341.3 nm, with no obvious blue shift, which indicates that the A20L peptide did not strongly interact with the eukaryotic cell membrane. By contrast, when the A20L interacted with the PC/PG liposomes, the maximum emission wavelength of tryptophan fluorescence was 315.7 nm was accomplished by a shift to blue color, suggesting that the tryptophan moved into the hydrophobic core of the membrane and could strongly interact with the prokaryotic membrane (Fig. 5a, Table 3).

**Table 3:** Tryptophan fluorescence emission maxima of A20L in different liposome models

Peptide	Liposome model	Wavelength (nm)	Blue shift (nm)	Intensity
A20L	HEPES	346.7	–	–
	PC/Chol = 8:1	341.3	5.4	36.855
	PC/PG = 7:3	315.7	31.0	220.809

The water-soluble fluorescent KI quencher was used to determine the degree of A20L interaction with prokaryotic and eukaryotic membranes as it is able to reduce the tryptophan fluorescence in the solution (Fig. 5b). The K*sv* value of A20L was lower when added to PC/PG and PC/Chol liposomes, suggesting that the antimicrobial peptide A20L is deeper inserted into the prokaryotic membrane, and, consequently, exhibit stronger interaction specificity with the prokaryotic membrane than with the eukaryotic one. These results are consistent with the findings from MIC, MHC and therapeutic index.

## Discussion

Through the use of fusion tags, the toxicity of AMPs to the host cells can be reduced and their resistance to intracellular protease degradation increased, thereby improving their production yield [9, 12]. Fusion tags can be classified into aggregation-promoting carriers and solubility-enhancing carriers. The PurF fragment, PaP3.30 and KSI are aggregation-promoting carriers. AMP fused with aggregation-promoting carriers was shown to be toxic to host bacterial cells [9, 22]. Thioredoxin (17 kDa) and glutathione-S-transferase (26 kDa) are solubility-enhancing carriers, but their large molecular weight indirectly led to low AMP yields [23–25]. The Ub-tag and SUMO1/2/3/4-tags are small and thus are useful for increasing the proportion of AMPs in fusion proteins, thereby increasing the yield of AMPs. Currently, NZ17074, lacticin Q and cecropin AD have been expressed successfully in *E. coli*, *B. subtilis* and *P. pastoris* by fusion to the SUMO-tag [26–28]. However, yeast cells are most commonly reported as the source of SUMO-tagged fusion peptides [11], while Ub-tagged and human SUMO-tagged fusion peptides are rarely reported. The use of a SUMO4-tag and Ub-tag for AMP expression has not yet been reported previously. Herein, we explored the expression of A20L tagged with an Ub-tag and SUMO1/2/3/4-tags to optimize the expression approach of AMP production. Our findings indicate that the expression yield was the highest for the Ub-tag fusion, and the SUMO1-tag was superior to the SUMO2/3/4-tags. It was interesting to see that all the human Ub-tag and SUMO1/2/3/4-tags were effective as fusion tags of AMPs; moreover, the Ub-tag not only promoted the soluble expression of the AMP, but also produced a higher yield than the human SUMO1/2/3/4-tags. The enhancement of both solubility and yield of the peptide A20L obatained by using Ub-tag and human SUMO1/2/3/4-tag systems should enhance the large-scale production of AMPs. Future work will approach further research to optimize the fusion tags in order to increase AMPs production.

AMPs can act by inducing membrane injury or intracellular injury [29]. The antibacterial activity of α-helical AMPs depends on their helicity, positive charge and hydrophobicity, *etc*. After interaction with bacterial cell membranes carrying negative charges, the secondary structure of AMPs is induced to become α-helical in nature, destroying the membrane integrity, affecting the electrochemical potential and ionic balance of the cell membrane, resulting in leakage of the cell contents, and finally causing bacterial death [29]. A20L peptides contain hydrophobic and positively charged amino acids [13]. Circular dichroism experiments have shown that the secondary structure of A20L adopted an α-helical from a random coil structure after the interaction with hydrophobic solvents that mimick the hydrophobic environment of cell membranes. In the bacterial membrane permeabilization experiments, A20L peptide caused rapid damage to the outer membrane of Gram-negative bacteria, allowing NPN to insert into the hydrophobic environment, thereby yielding increased fluorescence. In addition, after the damage to the bacterial inner membrane by A20L, β-galactosidase was released from the cells and reacted with the substrate, ONPG, suggesting that the bacterial membrane is the target of A20L peptide.

Bacterial and eukaryotic cells exhibit many differences in the structure and function of their membranes. Prokaryotic cells with a large amount of negative charges are mainly composed of phosphatidylglycerol, cardiolipin, and phosphatidylserine. In contrast, eukaryotic cells have zwitterionic phospholipids, including phosphatidylethanolamine, phosphatidylcholine, cholesterol, and sphingomyelin. Different membrane lipid compositions play a crucial role in the specificity of AMPs [30]. In this regard, we prepared liposomes containing different phospholipids to mimic prokaryotic and eukaryotic membranes. Tryptophan fluorescence and KI quenching experiments showed a stronger interaction between the A20L peptide and the prokaryotic membrane. The mechanisms used to describe the interaction of AMPs with bacterial cell membranes include the “carpet” model, “barrel-stave” model, “toroidal-pore” model and “aggregate channel” model [5]. We suggest that the “membrane discrimination mechanism” could well describe how α-helical AMPs interact with cell membranes as well as the specificity of AMPs [16]. The mechanism depends upon the difference in membrane composition between prokaryotic and eukaryotic cells. A20L forms pores/channels in the hydrophobic core of the eukaryotic bilayer by bundles of amphipathic α-helices producing an aqueous pore, it caused the hemolysis of human red blood cells; the mechanism of A20L with eukaryotic cells is a “barrel-stave” mechanism. The positive charge residues of A20L interact with the negatively charged phospholipid in prokaryotic cells, A20L kills prokaryotic cells by the “carpet” mechanism.

## Conclusions

In conclusion, both the Ub-tag and SUMO1/2/3/4-tags may be useful for the fusion expression of α-helical AMPs on an industrial scale. Ub-tag was proved as the most efficient approach for the expression of α-helical antimicrobial peptide A20L. Application of fusion tags for production of AMPs would help to reduce costs and would be widely applicable for use with a variety of AMPs. The MIC and MHC values indicated that the A20L peptide obtained from the fusion expression showed good antibacterial and low hemolytic activities. The results from the interaction of the A20L peptide with liposomes indicated that the membrane discrimination mechanism may be suitable for explaining the mechanism of action of α-helical AMPs.

## Additional file

Additional file 1: Table S1. DNA and amino acid sequences of Ub-A20L and SUMO 1/2/3/4-A20L. **Table S2.** Primer sequences for the A20L genes cloned and fused to SUMO and Ub. (PDF 63 kb)

## Abbreviations

Ub-tag: Ubiquitin-tag
SUMO-tag: Small ubiquitin-related modifier-tag
MHC: Minimal hemolytic concentration
AMP: Antimicrobial peptide
MIC: Minimal inhibitory concentrations
CD: Circular dichroism
PMSF: Phenylmethanesulfonyl fluoride
TFE: 2,2,2-Trifluoroethanol
NPN: 1-N-Phenylnaphthylamine
ONPG: ο-Nitrophenyl β-D-galactopyranoside
TI: Therapeutic index
BCA: Bicinchoninic Acid
PG: L-α-phosphatidyl-dl-glycerol
PC: L-α-phosphatidylcholine
Chol: Cholesterol
KI: Potassium iodide
