# Supplementary material for: Prokaryotic expression and mechanism of action of α-helical antimicrobial peptide A20L using fusion tags
Source: BMC Biotechnol. 2015 Aug 5;15:69. doi: 10.1186/s12896-015-0189-x (PMC4523955; doi:10.1186/s12896-015-0189-x)
Supplement: Additional file 1: Table S1. — DNA and amino acid sequences of Ub-A20L and SUMO 1/2/3/4-A20L. Table S2. Primer sequences for the A20L genes cloned and fused to SUMO and Ub. (PDF 63 kb) [file 12896_2015_189_MOESM1_ESM.pdf]

Supplementary Data

Table S1 DNA and amino acid sequences of Ub-A20L and SUMO 1/2/3/4-A20L

Ub-A20L:
ATGGGCAGCAGCCATCATCATCATCATCACAGCAGCGGCCTGGTGCCGCGCGGCAGCC
ATATGCAGATCTTTGTGAAGACCCTCACTGGCAAAACCATCACCCTTGAGGTCGAGCC
CAGTGACACCATTGAGAATGTCAAAGCCAAAATTCAAGACAAGGAGGGTATCCCACCT
GACCAGCAGCGTCTGATATTTGCCGGCAAACAGCTGGAGGATGGCCGCACTCTCTCAG
ACTACAACATCCAGAAAGAGTCCACCCTGCACCTGGTGTTGCGCCTCCGCGGTGGATC
CAAATGGAAATCTTTCCTGAAAACCTTCAAATCTGCTAAAAAAACCGTTCTGCACACC
CTGCTGAAAGCTATCTCTTCTTGAGAATTC
MGSSHHHHHHSSGLVPRGSHMQIFVKTLTGKTITLEVEPSDTIENVKAKIQDKEGIPPDQQ
RLIFAGKQLEDGRTLSDYNIQKESTLHLVLRLRGGSKWKSFLKTFKSAKKTVLHTLLKAIS
S
SUMO1-A20L:
ATGGGCAGCAGCCATCATCATCATCATCACAGCAGCGGCCTGGTGCCGCGCGGCAGCC
ATATGATGTCTGACCAGGAAGCTAAACCGTCTACCGAAGACCTGGGTGACAAAAAAGA
AGGTGAATACATCAAACTGAAAGTTATCGGTCAGGACTCTTCTGAAATCCACTTCAAA
GTTAAAATGACCACCCACCTGAAAAAACTGAAAGAATCTTACTGCCAGCGTCAGGGTG
TACCGATGAACTCTCTGAGGTTCCTGTTCGAAGGCCAGCGTATCGCTGACAACCACAC
CCCGAAAGAACTGGGTATGGAAGAAGAAGACGTTATCGAAGTTTACCAGGAACAGAC
CGGTGGTGGATCCAAATGGAAATCTTTCCTGAAAACCTTCAAATCTGCTAAAAAAACC
GTTCTGCACACCCTGCTGAAAGCTATCTCTTCTTGAGAATTC
MGSSHHHHHHSSGLVPRGSHMMSDQEAKPSTEDLGDKKEGEYIKLKVIGQDSSEIHFKVK
MTTHLKKLKESYCQRQGVPMNSLRFLFEGQRIADNHTPKELGMEEEDVIEVYQEQTGGG
SKWKSFLKTFKSAKKTVLHTLLKAISS

SUMO2-A20L:
ATGGGCAGCAGCCATCATCATCATCATCACAGCAGCGGCCTGGTGCCGCGCGGCAGCC
ATATGATGGCTGACGAAAAACCGAAAGAAGGTGTTAAAACCGAAAACAACAACCACA
TCAACCTGAAAGTTGCTGGTCAGGACGGTTCTGTTGTTCAGTTCAAAATCAAACGTCC
CACCCCGCTGTCTAAACTGATGAAAGCTTACTGCGAACGTCAGGGTCTGTCTATGCGTC
AGATCAGGTTCCGTTTCGACGGCCAGCCGATCAACGAAACCGACACCCCGGCTCAGCT
GGAAATGGAAGACGAAGACACTATAGACGTCTTCCAGCAGCAGACGGGTGGATCCAA
ATGGAAATCTTTCCTGAAAACCTTCAAATCTGCTAAAAAAACCGTTCTGCACACCCTG
CTGAAAGCTATCTCTTCTTGAGAATTC
MGSSHHHHHHSSGLVPRGSHMMADEKPKEGVKTENNNHINLKVAGQDGSVVQFKIKRP
TPLSKLMKAYCERQGLSMRQIRFRFDGQPINETDTPAQLEMEDEDTIDVFQQQTGGSKWK
SFLKTFKSAKKTVLHTLLKAISS

SUMO3-A20L:
ATGGGCAGCAGCCATCATCATCATCATCACAGCAGCGGCCTGGTGCCGCGCGGCAGCC

ATATGATGTCTGAAGAAAAACCGAAAGAAGGTGTTAAAACCGAAAACGACCACATCA
ACCTGAAAGTTGCTGGTCAGGACGGTTCTGTTGTTCAGTTCAAAATCAAACGTCACAC
CCCGCTGTCTAAACTGATGAAAGCTTACTGCGAACGTCAGGGTCTGTCTATGCGTCAGA
TCAGGTTCCGTTTCGACGGCCAGCCGATCAACGAAACCGACACCCCGGCTCAGCTGG
AAATGGAAGACGAAGACACTATAGACGTCTTCCAGCAGCAGACGGGTGGATCCAAAT
GGAAATCTTTCCTGAAAACCTTCAAATCTGCTAAAAAAACCGTTCTGCACACCCTGCT
GAAAGCTATCTCTTCTTGAGAATTC
MGSSHHHHHHSSGLVPRGSHMMSEEKPKEGVKTENDHINLKVAGQDGSVVQFKIKRHTP
LSKLMKAYCERQGLSMRQIRFRFDGQPINETDTPAQLEMEDEDTIDVFQQQTGGSKWKSF
LKTFKSAKKTVLHTLLKAISS

SUMO4-A20L:
ATGGGCAGCAGCCATCATCATCATCATCACAGCAGCGGCCTGGTGCCGCGCGGCAGCC
ATATGATGGCTAACGAAAAACCGACCGAAGAAGTTAAAACCGAAAACAACAACCACA
TCAACCTGAAAGTTGCTGGTCAGGACGGTTCTGTTGTTCAGTTCAAAATCAAACGTCA
GACCCCGCTGTCTAAACTGATGAAAGCTTACTGCGAACCGCGTGGTCTGTCGGTTAAG
CAGATCAGGTTCCGTTTCGGTGGCCAGCCGATCTCTGGTACCGACAAACCGGCTCAGC
TGGAAATGGAAGACGAAGACACTATAGACGTGTTCCAGCAACCGACCGGTGGATCCA
AATGGAAATCTTTCCTGAAAACCTTCAAATCTGCTAAAAAAACCGTTCTGCACACCCT
GCTGAAAGCTATCTCTTCTTGAGAATTC
MGSSHHHHHHSSGLVPRGSHMMANEKPTEEVKTENNNHINLKVAGQDGSVVQFKIKRQT
PLSKLMKAYCEPRGLSVKQIRFRFGGQPISGTDKPAQLEMEDEDTIDVFQQPTGGSKWKSF
LKTFKSAKKTVLHTLLKAISS

Table S2 Primer sequences for the A20L genes cloned and fused to SUMO and Ub[a]

Primer Name	Primer Sequence
5'-Ub-A20L	5' CGC**GGATCC**AAATGGAAATCTTTCCTGAAAACCTTC 3'
5'-SUMO1-A20L	5' CGC**CATATG**ATGTCTGACCAGGAAGCTAA 3'
5'-SUMO2-A20L	5' CGC**CATATG**ATGGCTGACGAAAAACCGAAAGA 3'
5'-SUMO3-A20L	5' CGC**CATATG**ATGTCTGAAGAAAAACCGAAAG 3'
5'-SUMO4-A20L	5' CGC**CATATG**ATGGCTAACGAAAAACC 3'
3'-Ub-A20L	5' CCG**GAATTC**TCAAGAAGAGATAGCTTTCAGCAG 3'
3'-SUMO1-A20L	5' CCG**GAATTC**TCAAGAAGAGATAGCTTTCAGCAG 3'
3'-SUMO2-A20L	5' CCG**GAATTC**TCAAGAAGAGATAGCTTTCAGCAG 3'
3'-SUMO3-A20L	5' CCG**GAATTC**TCAAGAAGAGATAGCTTTCAGCAG 3'
3'-SUMO4-A20L	5' CCG**GAATTC**TCAAGAAGAGATAGCTTTCAGCAG 3'

[a] The restriction sites are shown in bold.

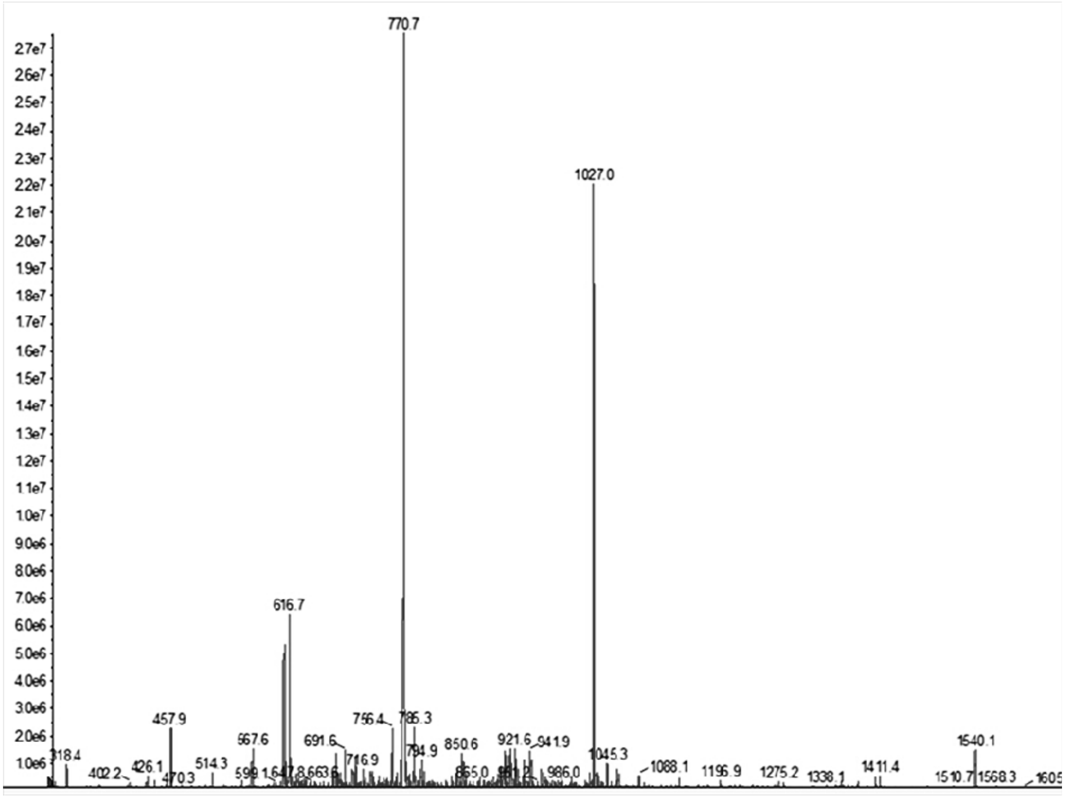

Figure S1 Mass spectroscopy of the antimicrobial peptide A20L
